# Increased malondialdehyde concentration and reduced total antioxidant capacity in aqueous humor and blood samples from patients with glaucoma

**Published:** 2013-08-07

**Authors:** Carlo Nucci, Donato Di Pierro, Chiara Varesi, Elena Ciuffoletti, Rossella Russo, Rocco Gentile, Claudio Cedrone, Maria Dolores Pinazo Duran, Massimiliano Coletta, Raffaele Mancino

**Affiliations:** 1 Ophthalmology Unit, Department of Exp. Medicine and Surgery, Tor Vergata University of Rome, Italy; 2 Department of Clinical Science and Translational Medicine, Tor Vergata University of Rome, Rome, Italy; 3 Department of Pharmacobiology, University of Calabria, Cosenza, Italy; 4 Ophthalmic Research Unit, “Santiago Grisolia,” University Hospital Dr. Peset, Valencia, Spain

## Abstract

**Purpose:**

To evaluate the levels of malondialdehyde (MDA) and total antioxidant capacity (TAC) in the blood and aqueous humor of glaucomatous and nonglaucomatous patients. To measure the adenosine triphosphate/adenosine diphosphate/adenosine monophosphate (ATP/ADP/AMP) concentration as a biomarker of the blood energy charge potential.

**Methods:**

We examined 40 consecutive patients with primary open-angle glaucoma scheduled for cataract surgery. Twenty-six age-matched subjects scheduled for cataract surgery were enrolled as a control group. Blood and aqueous humor samples were collected at the time of surgery. MDA concentrations and blood nucleotides were measured with high-performance liquid chromatography. The TAC of the samples was estimated with the oxygen-radical absorbance capacity method.

**Results:**

Blood and aqueous humor MDA levels in glaucoma patients (respectively, 0.976±0.370 and 0.145±0.065 μmol/ml) were significantly increased (p<0.001 for both) over those of the control group (respectively, 0.454±0.395 and 0.060±0.039 μmol/ml). In contrast, the control group presented significantly higher TACs than did the glaucoma group in both the blood (control: 2.681±1.101 and glaucoma: 1.617±0.674 μmol Trolox Equi/g; p<0.001) and aqueous humor (control: 0.963±0.302 and glaucoma: 0.788±0.346 μmol Trolox Equi/g; p=0.039). The control group (0.869±0.037) exhibited statistically significant (p<0.001) higher values of blood adenosine triphosphate/adenosine diphosphate (ATP-ADP) levels than did the glaucoma group (0.791±0.037).

**Conclusions:**

Our data further support the hypothesis that oxidative stress and decreased antioxidant defenses are involved in glaucoma. High-performance liquid chromatography appears to be an effective and sensitive method to detect altered levels of oxidative stress markers in glaucoma patients.

## Introduction

Glaucoma is a progressive optic neuropathy that affects nearly 90 million people worldwide, and is the leading cause of irreversible blindness [1-3]. It is now considered a multifactorial disease in which high intraocular pressure (IOP) is the most important known risk factor for the development of glaucomatous optic nerve damage [4]. Increasing evidence indicates that oxidative stress plays a key role in the pathogenesis of primary open-angle glaucoma (POAG) [5,6]. The primary POAG-related damage is in fact constituted by the occurrence of degenerative phenomena affecting the sclerocorneal trabecular meshwork (TM), and a large number of experimental studies support the hypothesis that the progressive loss of TM cells in glaucomatous patients may be ascribed to the long-term effects of oxidative damage induced by free radicals [7,8]. Moreover, it has been demonstrated that the most severe TM alterations in POAG occur in the anatomic layers in closest contact with the anterior chamber [8]. Under physiologic conditions, free radicals are actively neutralized by the antioxidant system [9], naturally present at the level of the aqueous humor. The alteration of this equilibrium may give rise to the progressive accumulation of oxidative damage in TM and, consequently, to the increasing of intraocular pressure. However, apart from these interesting studies, few research studies have evaluated the oxidative and antioxidant capacities and the energy status of body fluids in patients with glaucoma. To address this issue, we analyzed samples of blood and aqueous humor from glaucoma patients and nonglaucomatous controls. Each specimen was subjected to high-performance liquid chromatography (HPLC) to determine the levels of malondialdehyde (MDA) produced during phospholipid peroxidation [10,11] and the activation of the arachidonate cycle [12]; MDA is widely regarded as a marker of peroxidative damage to cell membranes that is induced by physical and/or chemical oxidative stress [13]. The total antioxidant capacity (TAC) of each sample was also determined, using the oxygen-radical absorbance capacity (ORAC) method and using ATP, ADP, and AMP levels detected with HPLC to identify them as a biomarker of tissue energy status and, eventually, their energy charge potential (ECP). The application of HPLC produces findings that indicate the importance of oxidative stress in glaucoma, since HPLC minimizes the risk of metabolite concentrations being altered during measurement. It also provides clear, reliable, reproducible values on peroxidative damage and energy metabolism, both of which are fundamental parameters in the study of degenerative eye disease.

## Methods

The study was approved by the institutional review board of the University Hospital Tor Vergata. The study followed the tenets of the Declaration of Helsinki. All participants provided informed consent after explanation of the nature and possible consequences of the study. The study group consisted of 40 consecutive patients with primary open-angle glaucoma (22 males and 18 females; mean±SD age: 75.3±9.1 years) recruited from the Ophthalmology Unit of the Tor Vergata University of Rome Medical Hospital. Inclusion criteria for glaucoma patients were the following that the vertical cup/disk ratio was >0.5 and that there were typical glaucomatous defects at the visual field (as determined by the 24–2 Sita-standard program of the Humphrey Visual Field Analyzer, with IOP measured with Goldmann applanation tonometry controlled only by use of topical medical therapy). None of the glaucoma patients had previous incisional or laser surgery.

For comparison purposes, we also enrolled a control group consisting of 26 age-matched nonglaucomatous subjects (14 males and 12 females, mean age 74.0±8.3 years).

### Glaucoma and control subjects were scheduled for cataract surgery

Glaucoma and control candidates were excluded from the study for any of the following reasons: smoking, diabetes, liver disease, severe nephropathy, cancer, collagen diseases, acute or chronic infections, fever, congestive heart failure, use of oral antioxidant supplements, and the presence of any other eye disease, such as age-related macular degeneration and diabetic retinopathy.

### Sample collection

Samples of blood and aqueous humor were collected from all participants on the day of surgery. Venous blood samples were drawn after an overnight fast. The samples were placed on ice and centrifuged within 1 h (3500 ×g at 4 °C for 15 min), and the supernatants were stored at −20 °C until analyzed. Aqueous humor samples (0.1–0.2 ml) were rapidly collected at the beginning of cataract surgery with a 27-gauge needle on a tuberculin syringe. Special care was taken to avoid blood contamination. Samples were immediately cooled and stored at −70 °C.

### Quantitative analysis of malondialdehyde and energy state

Blood and aqueous humor were collected in sterile tubes. Ice-cold 1.2 M HClO_4_ (1:2, w/w) was added to the blood samples to deproteinize erythrocytes. All samples were centrifuged at 20,690×g for 10 min at 4 °C, neutralized by adding 5 M K_2_CO_3_ in ice, filtered through a 0.45 μm Millipore-HV filter (Merck Millipore, Merck KGaA, Darmstadt, Germany), and subjected to HPLC. The ion-pairing method was used for simultaneous direct determination of MDA and adenine nucleotide levels in 100 μl of a perchloric acid extract of each sample [11,12]. We used a Vydac 250×4.6 mm, 5 μm particle-size column with its own guard column (Eka Chemicals AB, Bohus, Sweden) and tetrabutylammonium hydroxide as the ion-pairing reagent. Briefly, metabolites were separated by creating a step gradient (adapted to the column size [11,12] with two buffers: buffer A [10 mM tetrabutylammonium hydroxide, 10 mM KH_2_PO_4_, 0.25% methanol pH 7.00] and buffer B [2.8 mM tetrabutylammonium hydroxide, 100 mM KH_2_PO_4_, 30% methanol pH 5.50]). The gradient was as follows: 10 min, 100% with buffer A; 3 min, 90% with buffer A; 10 min, 70% with buffer A; 12 min, 55% with buffer A; 15 min, 45% with buffer A; 10 min, 25% with buffer A; 5 min, 0% with buffer A. A flow rate of 1.2 ml/min was maintained throughout each run, and the column temperature was maintained at 23 °C with the aid of water-jacketed glassware. The HPLC apparatus consisted of a Surveyor LC Pump (ThermoFinnigan Italia, Rodano, Milan, Italy) connected to a Surveyor PDA Detector (ThermoFinnigan Italia) with a wavelength range of 200–300 nm. Data were acquired and analyzed with the ChromQuest program (ThermoQuest, Milan, Italy). Areas, retention times and absorbance spectra of the peaks of sample chromatograms were compared with those of freshly prepared ultrapure standards to determine the concentration of the various compounds at 267 nm (the upper limit of the MDA absorbance spectrum) and identify different metabolites. Hemoglobin and the percentage of hemolysis were calculated with standard techniques [14] in a Jasco-685 double-beam spectrophotometer (Jasco Europe, Lecco, Italy).

The blood energy-state levels were determined by employing ATP, ADP, and AMP detected by HPLC, and the ECP was calculated according to the following formula:

ECP=ATP+0.5 ADP/∑NT

where 

∑NT=ATP+ADP+AMP

is the sum of the adenine nucleotide levels [15].

### Oxygen radical absorbance capacity assay

The ORAC assay is based on the dose- and time-dependent decrease in the fluorescence intensity of β-phycoerythrin (β-PE) when oxidized by oxygen radicals [13]. It measures the antioxidant capacity of a substance—blood, vitreous, and aqueous humor in this case—in terms of its ability to inhibit or delay β-PE peroxidation.

Our assay was performed with the original method described by Cao et al. [16], with a few modifications [17,18]. AAPH [2,2'-Azobis(2-aminopropane)dihydrochloride] purchased from Polyscience (Warrington, PA) was used as the free-radical generator, and β-PEl was purchased from Sigma-Aldrich (Sigma-Aldrich Co, St. Louis, MO). The final reaction mixture (2 ml) contained 1.750 ml of 75 μM phosphate buffer (pH 7.0) plus 0.100 ml of one of the following: 20 μM Trolox (6-hydroxy-2,5,7,8-tetramethyl-2-carboxylic acid), which was used as the standard; body fluid sample (blood, aqueous humor, or vitreous); or buffer alone (used as the blank). Beta-phycoerythrin (0.100 ml of a 34 mg/l solution) was placed in each well, and the oxidant reaction was started by adding 160 mM AAPH (0.050 ml per well). Beta-PE fluorescence was measured with a Varian Cary Eclipse Fluorescence Spectrofotometer (Varian Ltd., Madrid, Spain) at λ=546 nm (λ excitation) and λ=573 nm (λ emission). Measurements were made every 2.5 min at 37 °C for 1 h or until the fluorescence variation dropped below 2%. The ORAC of the sample was expressed as Micromol Trolox Equivalents/g and calculated as [(As–Ab)/(At–Ab)]ka, where As is the area under the curve (AUC) of β-PE in the sample, calculated with the Origin 2.8 Integration Program (MicroCal Software, LLC, Northhampton, MA), At is the AUC of the Trolox, Ab is the AUC of the control, k is the dilution factor (1:500 for the blood, 1: 100 for aqueous humor), and a is the concentration of the Trolox in mmol/l.

### Statistical analysis

For continuous variables, data were compared between groups using the unpaired Student’s t-test. For categorical variables, comparison between groups was done using the chi-square test. Correlations of the independent variables age and sex with the dependent variables studied (blood or aqueous MDA, blood or aqueous TACs, blood ECP) were evaluated with the Spearman nonparametric test. Significance was set at p<0.05.

## Results

The mean±SD age of the control group (74.0±8.3 years) and of the glaucoma group (75.3±9.1 years) did not differ statistically (Student’s t-test p=0.559). The male/female ratio of the control group (14/12=1.17) and of the glaucoma group (22/18=1.22) did not differ statistically (chi-square p=0.872). Table 1 reports the MDA levels in the blood and aqueous humor of the controls and glaucoma patients. Blood and aqueous humor MDA levels in glaucoma patients were significantly increased over those of the control group (p<0.001 for both groups). In contrast, the control group presented significantly higher TACs than did glaucoma group in both blood (p<0.001) and aqueous humor (p=0.004), as shown in Table 2. The ECP, as defined by Equation 1, was measured only in the blood samples. The control group (mean±SD: 0.869±0.037) exhibited statistically significant (Student’s t-test p<0.001) higher values than did the glaucoma group (mean±SD: 0.791±0.037). The independent variables, age and sex, did not correlate (Spearman test p>0.05) with any of the dependent variables studied (blood or aqueous MDAs, blood or aqueous TACs, blood ECP; Table 3).

**Table 1:** Malondialdehyde (MDA) levels in the blood, and aqueous humor of glaucoma patients and controls.

**Samples**	**Control group** **(26 subjects)**	**Glaucoma group** **(40 subjects)**
**Blood**	0.454±0.395	0.976±0.370
**Aqueous**	0.060±0.039	0.145±0.065

The levels of MDA were measured by High Performance Liquid chromatography in the blood, and aqueous humor of the control, and glaucoma groups. Blood and aqueous humor MDA levels in glaucoma patients were significantly increased over those of control group (both, p<0.001). Data are expressed in μmol/ml and represent mean ± standard deviation. Student *t* test analysis was used.

**Table 2:** Total antioxidant capacity (TAC) in the blood, and aqueous humor of control and glaucoma patients.

**Samples**	**Control group** **(26 subjects)**	**Glaucoma group** **(40 subjects)**
**Blood**	2.681±1.101	1.617±0.674
**Aqueous**	0.963±0.302	0.788±0.346

TAC was measured by the oxygen radical absorbance capacity assay in the blood, and aqueous humor of controls and glaucoma patients. The control group displayed significantly higher TAC levels than glaucoma group in both the blood (p<0.001) and aqueous humor (p=0.004). Data are expressed in μmol Trolox Equi/g and represent mean ± standard deviation. Student *t* test analysis was used.

**Table 3:** The independent variables age and sex did not correlate with all the dependent variables studied (age, all variables, p>0.10; sex, malondialdehyde (MDA) aqueous/ total antioxidant capacity (TAC) blood, p>0.05; sex, MDA blood/energy charge potential (ECP)/TAC aqueous, p>0.10).

**Variables**	**MDA**		**ECP**	**TAC**	
	**Blood**	**Aqueous**	**Blood**	**Blood**	**Aqueous**
**Age**	0.148	0.194	0.222	0.043	−0.009
**Sex**	0.052	0.286	0.097	0.279	0.136

Data represent r values found during the Spearman nonparametric statistical test analysis.

## Discussion

In the present study, we attempted to characterize the oxidative stress and total antioxidant capacities of blood and aqueous humor in glaucoma patients. Oxidative stress has long been involved in the pathogenesis of cataract [19]. This is why we analyzed glaucoma patients scheduled for cataract surgery with IOP controlled by the only use of topical medical therapy: in this way, the values of the oxidative stress and the antioxidant capacity were comparable to those of the cataract group.

Our results suggest that glaucoma patients had significantly higher levels of MDA and lower levels of TAC in the blood and aqueous humor. Oxidants are highly reactive compounds with half-lives of a few seconds, and this seriously hinders their measurement, in vivo, in the eye [20]. However, specific biomarkers have been investigated to evaluate oxidative stress, including the breakdown products of peroxidized polyunsaturated fatty acids, such as MDA, which has proven to be both sensitive and reliable for this purpose [18]. Ion-pairing HPLC with tetrabutylammonium allows the simultaneous measurement of all nucleotides and corresponding deoxynucleosides, with no chemical manipulation of samples other than perchloric acid deproteinization. This approach minimizes the risk that metabolite concentrations will be altered and provides clear, reliable, reproducible values on peroxidative damage and energy metabolism, both of which are fundamental parameters in the study of degenerative eye disease [21,22].

Increased levels of oxidative agents have been found in the aqueous humor of glaucomatous patients, and in a recent study, MDA concentrations measured spectrophotometrically using a thiobarbituric acid-reacting substrate were found to be higher in the aqueous humor of glaucoma patients than in that of healthy controls [23]. Our study is the first to use HPLC to explore this issue, and our findings confirm this previous report on aqueous humor MDA levels and extend the observation to the serum. This suggests that high serum levels of MDA in glaucoma patients may reflect similar increases at the level of the aqueous humor.

It has been proposed that oxidative stress can damage the cells of the trabecular meshwork [6]. The TM, which forms the major route for the aqueous outflow from the anterior chamber, contains a sophisticated defense mechanism against reactive oxygen species (ROS) [8]. The antioxidant status of a biologic sample could be regarded as an indicator of oxidative stress: a decrease in the antioxidant capacity of tissues and body fluids may be the consequence of increased oxidative processes. Data from Ferreira et al. [24] suggested that oxidative stress could have a role in the pathogenesis of POAG and that ROS might lead to an induction of antioxidant enzymes and might contribute to decreasing reactive antioxidants. Their results accord with our study, which shows that the total antioxidant activity was significantly decreased in glaucoma patients, both at level of the aqueous humor and blood. It must be assumed that an increase in antioxidant activity can be the initial step of the oxidation process. However, a decreased antioxidant capacity of tissues and fluids may be the consequence of long-lasting oxidative changes. Because it is relatively difficult to measure individual antioxidants separately, specific assays have been designed to measure the overall oxygen radical-scavenging capacity of fluid samples. The ORAC assay has been found to provide a good index of the total antioxidant capacity [12,18].

Finally, the blood of our glaucoma patients exhibited significantly reduced energy charge potentials, compared with controls. The ATP/ADP level ratio represents the balance between energy-producing and energy-consuming reactions, and decreased values generally reflect insufficient production of ATP via the oxidative and phosphorylative activities of the mitochondria. Our finding indicates for the first time that in glaucoma patients, the blood energy supply is deficient and its reduction may be a primary event in the development of the disease.

On the whole, these data indicate that altering the redox state and energy potential contribute to the development of glaucoma, although the mechanisms by which oxidative stress triggers this event have not been fully elucidated. Oxidative stress has been shown to induce cell death by targeting the mitochondria directly, which are both a major endogenous source and target of ROS [25]. Reactive oxygen species have also been involved in apoptosis of the retinal ganglion cells [26,27].

A large number of mechanisms are implicated in neuronal death in glaucoma and include glutamate excitotoxicity, nerve growth factor deprivation, ischemia, and autoimmune and oxidative damage [26,28,29]. Therapeutic intervention directed at a process mediated by ROS might be effective in preventing TM damage [30], but might also be neuroprotective of retinal ganglion cell (RGC) [31-33].

In conclusion, this study demonstrates that HPLC is an effective, sensitive method for detecting altered levels of oxidative stress markers in glaucoma patients. Our results strongly suggest that an increase in free radical formation and a decrease in the antioxidant defense mechanism may play a role in the pathogenesis of POAG. We propose that the determination of blood oxidative stress, TAC, and ECP may be assessed by using HPLC to help understand individuals’ vulnerability to the disease.
